# Expanding the Role of Ultrasound for the Characterization of Renal Masses

**DOI:** 10.3390/jcm11041112

**Published:** 2022-02-19

**Authors:** Eduard Roussel, Riccardo Campi, Daniele Amparore, Riccardo Bertolo, Umberto Carbonara, Selcuk Erdem, Alexandre Ingels, Önder Kara, Laura Marandino, Michele Marchioni, Stijn Muselaers, Nicola Pavan, Angela Pecoraro, Benoit Beuselinck, Ivan Pedrosa, David Fetzer, Maarten Albersen, on behalf of the European Association of Urology (EAU) Young Academic Urologists (YAU) Renal Cancer Working Group

**Affiliations:** 1 Department of Urology, University Hospitals Leuven, 3000 Leuven, Belgium; maarten.albersen@uzleuven.be; 2 Unit of Urological Robotic Surgery and Renal Transplantation, Careggi Hospital, University of Florence, 50134 Firenze, Italy; riccardo.campi@unifi.it; 3 Department of Urology, San Luigi Gonzaga Hospital, University of Turin, 10043 Orbassano, Italy; danieleamparore@hotmail.it (D.A.); pecoraro416@gmail.com (A.P.); 4 Department of Urology, San Carlo Di Nancy Hospital, 00165 Rome, Italy; riccardobertolo@hotmail.it; 5 Department of Emergency and Organ Transplantation-Urology, Andrology and Kidney Transplantation Unit, University of Bari, 70121 Bari, Italy; u.carbonara@gmail.com; 6 Division of Urologic Oncology, Department of Urology, Istanbul University Istanbul Faculty of Medicine, 34093 Istanbul, Turkey; erdemselcuk1@gmail.com; 7 Department of Urology, University Hospital Henri Mondor, 94000 Créteil, France; alexandre.ingels@gmail.com; 8 Department of Urology, Kocaeli University School of Medicine, 41001 Kocaeli, Turkey; onerkara@yahoo.com; 9 Department of Medical Oncology, Fondazione IRCCS Istituto Nazionale dei Tumori, 20133 Milan, Italy; laura.lmarandino@gmail.com; 10 Department of Medical, Oral and Biotechnological Sciences, G. d’Annunzio University of Chieti, 66100 Chieti, Italy; mic.marchioni@gmail.com; 11 Department of Urology, Radboud University Medical Center, 6525 GA Nijmegen, The Netherlands; stijn.muselaers@radboudumc.nl; 12 Urology Clinic, Department of Medical, Surgical and Health Science, University of Trieste, 34127 Trieste, Italy; nicpavan@gmail.com; 13 Department of General Medical Oncology, University Hospitals Leuven, 3000 Leuven, Belgium; benoit.beuselinck@uzleuven.be; 14 Department of Radiology, University of Texas Southwestern Medical Center, Dallas, TX 75390, USA; ivan.pedrosa@utsouthwestern.edu (I.P.); david.fetzer@utsouthwestern.edu (D.F.); 15 Advanced Imaging Research Center, University of Texas Southwestern Medical Center, Dallas, TX 75390, USA; 16 Department of Urology, University of Texas Southwestern Medical Center, Dallas, TX 75390, USA

**Keywords:** ultrasound, ultrasonography, contrast-enhanced ultrasound, micro-Doppler, molecular ultrasound, renal mass, renal tumor, renal cell carcinoma

## Abstract

The incidental detection of renal masses has been steadily rising. As a significant proportion of renal masses that are surgically treated are benign or indolent in nature, there is a clear need for better presurgical characterization of renal masses to minimize unnecessary harm. Ultrasound is a widely available and relatively inexpensive real-time imaging technique, and novel ultrasound-based applications can potentially aid in the non-invasive characterization of renal masses. **Evidence acquisition:** We performed a narrative review on novel ultrasound-based techniques that can aid in the non-invasive characterization of renal masses. **Evidence synthesis:** Contrast-enhanced ultrasound (CEUS) adds significant diagnostic value, particularly for cystic renal masses, by improving the characterization of fine septations and small nodules, with a sensitivity and specificity comparable to magnetic resonance imaging (MRI). Additionally, the performance of CEUS for the classification of benign versus malignant renal masses is comparable to that of computed tomography (CT) and MRI, although the imaging features of different tumor subtypes overlap significantly. Ultrasound molecular imaging with targeted contrast agents is being investigated in preclinical research as an addition to CEUS. Elastography for the assessment of tissue stiffness and micro-Doppler imaging for the improved detection of intratumoral blood flow without the need for contrast are both being investigated for the characterization of renal masses, though few studies have been conducted and validation is lacking. **Conclusions:** Several novel ultrasound-based techniques have been investigated for the non-invasive characterization of renal masses. CEUS has several advantages over traditional grayscale ultrasound, including the improved characterization of cystic renal masses and the potential to differentiate benign from malignant renal masses to some extent. Ultrasound molecular imaging offers promise for serial disease monitoring and the longitudinal assessment of treatment response, though this remains in the preclinical stages of development. While elastography and emerging micro-Doppler techniques have shown some encouraging applications, they are currently not ready for widespread clinical use.

## 1. Introduction

There has been a steady increase in the incidental detection of small renal masses in the last several decades, at least in part due to the increased use of abdominal imaging [1]. This has contributed to an increase in the prevalence of renal cell carcinoma (RCC), which represents the most common renal malignancy. However, a significant proportion of renal masses are still being resected for the presumption of cancer, while otherwise being benign or indolent in nature [2]. Although renal mass biopsy is currently regarded as the gold standard for the presurgical diagnosis of benign or indolent disease, its widespread adoption has been hampered by a non-diagnostic rate of 10–15% and concerns about intratumoral heterogeneity [3,4].

Therefore, there is an apparent need for improved presurgical characterization of renal masses to avoid overtreatment. In this way, patients may be spared unnecessary interventions and the associated risk of perioperative morbidity or decreased renal function [5]. This may be particularly applicable for elderly or frail patients [6].

Currently, contrast-enhanced CT (CECT) is considered the standard of care for the assessment of solid renal tumors. Post-contrast enhancement and heterogeneity of a renal lesion are associated with malignancy and are used to distinguish between RCC subtypes to some extent, but with limited success [7,8,9,10,11,12,13]. Ultrasound is a non-invasive, widely used and readily available imaging technique offering real-time imaging without ionizing radiation. Moreover, the costs of ultrasound are significantly lower compared with computed tomography (CT) and magnetic resonance imaging (MRI), which might also benefit healthcare systems, particularly in lower-income countries. Conversely, ultrasound is often criticized due to its operator dependency. Conventional ultrasound is often sufficient to classify indeterminate renal masses as benign in the case of simple or minimally complex cystic masses, though it is not reliable to subtype more complex cystic and solid renal masses (i.e., with few and thin septae) [14,15]. Several novel approaches have been investigated to improve the diagnostic accuracy of ultrasound and expand its role in the characterization of renal masses.

We reviewed the current literature on the use of ultrasonography for the non-invasive characterization of renal masses with a focus on innovative techniques in order to critically assess its current role and potential future applications among other imaging modalities in this field.

Moreover, a considerable proportion of renal masses display equivocal imaging features and cannot reliably be distinguished as benign or malignant using conventional CT or MRI [7,8,12,13,16]. In light of these shortcomings, other approaches have been investigated, each with its strengths and limitations.

## 2. Evidence Acquisition

We performed a non-systematic, narrative review on the role of novel ultrasound-based techniques for the characterization of renal masses. A search of the Pubmed/MEDLINE database was conducted to identify original studies and review articles related to the use of ultrasound-based techniques in the evaluation of renal masses. Keywords included “kidney neoplasm”, “renal tumor”, “elastography”, “contrast-enhanced ultrasound”, “ultrasound” and “ultrasonography”, along with free-text or related and derivative terms. The search was conducted on 1 November 2021. In order to focus on innovative and modern techniques and applications, we initially included articles published in the last 10 years. Subsequently, additional manuscripts of interest were identified through a manual search of the reference lists of the retrieved articles. The final review represents an overview of novel ultrasonography-based applications for renal mass characterization and represents a consensus work.

## 3. Results

### 3.1. Contrast-Enhanced Ultrasound

**Figure 1:**
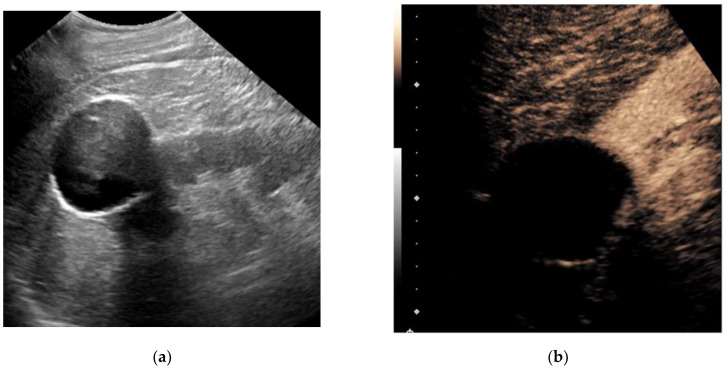
A 79-year-old man with a history of bladder cancer undergoing evaluation for hydronephrosis. Grayscale ultrasound image of the left kidney in the longitudinal orientation (**a**) shows an exophytic hypoechoic mass containing internal low-level echos. Following an intravenous injection of 1.8 cc Lumason ultrasound contrast, a contrast-enhanced ultrasound image focused at the upper pole (**b**) revealed the mass was completely non-enhancing (devoid of signal), which is diagnostic for a simple cyst. No further follow-up was necessary.

**Figure 2:**
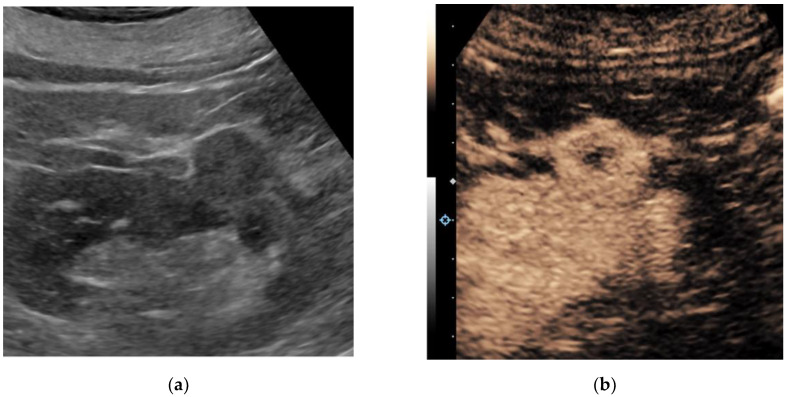
A 67-year-old man with multiple renal lesions, status post SBRT one year prior for contra-lateral RCC. Grayscale ultrasound image of the left kidney in longitudinal orientation (**a**) shows an isoechoic exophytic nodule. Following the intravenous administration of 1.0 cc Lumason ultrasound contrast, a contrast-enhanced ultrasound image (**b**) shows the nodule demonstrating predominantly solid avid enhancement relative to the adjacent renal cortex. A partial nephrectomy revealed clear cell renal cell carcinoma, grade 2.

Contrast-enhanced ultrasound (CEUS) is an emerging technique that addresses some of the limitations of non-enhanced grayscale and traditional Doppler ultrasound for the detection of vascularization within soft tissues. Intravenously administered ultrasound contrast agents consist of small particles: gas-filled cores encapsulated within biodegradable shells. These microbubbles, approximately the size of red blood cells, resonate non-linearly when insonnated by ultrasound. The unique signals from microbubbles can be separated from background tissues, allowing for the specific detection of blood flow within perfused tissues. CEUS can definitively confirm simple cysts via the lack of enhancement and may help to characterize solid renal lesions based on differences in lesional versus renal cortical perfusion [17,18]. Figure 1 and Figure 2 illustrate the performance of CEUS compared with traditional grayscale ultrasound by identifying enhancement within a renal mass. Despite the lack of radiation, low cost and highly favorable safety profile of ultrasound contrast agents, operator dependency and other technical factors may limit reproducibility [19,20].

CEUS has a distinct value in characterizing septations and mural nodules within cystic renal masses based on the presence or lack of enhancement, which is of specific interest for the equivocal Bosniak IIF and III cystic renal masses. However, specific definitions for CEUS imaging findings to predict the gradual increase in the risk of malignancy (i.e., equivalent to the Bosniak classification) are currently lacking [15]. According to several reports, the sensitivity and specificity of CEUS for malignancy in a cystic renal mass was comparable to CECT and MRI [19,21,22,23,24,25,26,27,28]. Additionally, CEUS was reported to perform at least as well as, if not better than, CECT and contrast-enhanced MRI (CEMR) in the classification of benign versus malignant renal masses [17,22,23,24,29,30,31]. Furrer et al. performed a meta-analysis on 1290 patients harboring 1342 cystic or solid renal masses, comparing the performance of CEUS to CECT and CEMR in the detection of benign versus malignant histology. They reported pooled sensitivity and specificity values for CEUS, CECT and CEMR of 96%, 90% and 96%, and 78%, 77% and 75%, respectively [17]. Their findings were in line with the findings of meta-analyses by Zhang et al., who compared CEUS to CECT in solid renal masses, and Zhou et al., who compared CEUS to CEMR in CRMs [22,31]. However, a low prevalence of certain benign tumor types (e.g., only 1% of renal masses were oncocytomas) likely reflects a considerable selection bias in these studies. Furthermore, it is unclear how many renal masses in these studies were excluded due to macroscopic fat seen during CT or MRI, which is virtually diagnostic of AML. They concluded that CEUS could be a valuable alternative to CECT in the evaluation of both solid as well as cystic renal masses, despite the heterogeneity across studies and the overall level of evidence being low [17]. An overview of key findings of CEUS studies is provided in Table 1.

Although quantitative CEUS features, such as the analysis of time–intensity curves, were shown to partially aid in the distinction of clear cell RCC (ccRCC), papillary RCC (papRCC) and chromophobe RCC (chrRCC) between each other and typical AML from RCCs, there is considerable overlap between imaging features of these renal tumor subtypes [32,33,34].

Moreover, both fat-poor AMLs (fpAML) and oncocytomas cannot be reliably distinguished from RCC on CEUS due to non-differing sonomorphological imaging features [23,32,35,36,37]. Therefore, CEUS is likely inadequate for subtyping all solid renal masses at this time.

### 3.2. Ultrasound Molecular Imaging

A highly innovative approach to ultrasound imaging involves the targeting and real-time, in vivo visualization of physiologic processes with molecular-specific imaging. Molecular-targeted microbubbles have been developed as an extension of CEUS [38,39]. This could be of particular interest in the metastatic setting, where the response to systemic therapy is currently determined by the change in tumor volume. However, changes in tumor physiology occur sooner than measurable tumor volume changes, which might allow for earlier assessment of tumor progression, response to systemic therapy and, ultimately, therapeutic decision making. Moreover, the use of molecular imaging techniques might better characterize features relating to intrinsic disease biology, such as angiogenesis, potentially leading to more individualized treatment decision making [40,41]. In a recent report, Rojas et al. studied ccRCC in a xenograft model of immunodeficient mice treated with the anti-angiogenic vascular endothelial growth factor receptor (VEGFR) tyrosine kinase inhibitor sunitinib [39]. They administered a microbubble contrast agent targeted to VEGFR-2 and subsequently imaged the tumors with CEUS after 1 week of treatment. They reported changes in VEGFR-2 expression at that time, as determined on ultrasound molecular imaging in the sunitinib-treated group, as opposed to changes in tumor volume, which only became apparent after 3 weeks. Moreover, after 1 week, response to therapy was detected in 92% of cases with ultrasound molecular imaging, whereas the detection rate was only 40% with volume measurements. Likewise, Ingels et al. studied the potential of ultrasound molecular imaging to track the response to sunitinib in a ccRCC mice xenograft model [38]. These mice, harboring ccRCC, were randomized between treatment with sunitinib and control and were injected with both non-targeted microbubbles and microbubbles targeting VEGFR-1 and follicle-stimulating hormone receptor (FSHR). Both the VEGFR-1 and FSHR signal enhancement were significantly lower in the sunitinib group at all times of treatment, while there was no significant difference between the two groups for the non-targeted microbubble ultrasound signal. Thus, they confirmed the potential of ultrasound molecular imaging for the longitudinal assessment of treatment response to sunitinib. However, despite its potential for serial monitoring of disease, as well as longitudinal assessment of disease response to systemic therapy, ultrasound molecular imaging is still in the very early phases of development and further research endeavors will determine whether these techniques can provide additional value in clinical practice.

### 3.3. Elastography

Equivalent to the use of palpation during physical examination, ultrasound elastography measures changes in tissue stiffness, which are often seen with diffuse parenchymal diseases and the associated changes in tissue architecture [42]. Strain elastography provides a qualitative or semi-quantitative assessment of tissue elasticity using external compression–decompression cycles from the ultrasound transducer. Shear-wave elastography (SWE) involves a quantitative assessment of tissue stiffness by measuring the propagation speed of generated shear waves through tissues. This technique does not require external compression by a transducer, relying instead on a high-amplitude push pulse (also known as acoustic radiation force impulse or ARFI), thus making it less operator dependent [43]. Strain elastography was shown to aid in the distinction of benign vs. malignant lesions, the distinction of RCC from AML and the distinction of RCC from transitional cell carcinoma [42,44,45]. SWE had potential value in the differentiation of ccRCC versus oncocytoma, ccRCC versus chrRCC or papRCC and pseudotumor from ccRCC or AML, though failed to differentiate between ccRCC and AML [43,46]. However, few studies have been conducted and these results lack validation, rendering clinical applications for renal mass characterization limited at this time. An overview of key findings of elastography studies is provided in Table 2.

### 3.4. Micro-Doppler Techniques

The presence of blood flow within a renal mass indicates solid tissue, as opposed to a renal cyst. The pattern of vascularity may help characterize indeterminate renal masses, such as differentiating malignancy from pseudomasses. Many companies are releasing novel micro-Doppler techniques with advanced clutter suppression. Some of these include Superb Micro-Vascular Imaging (Canon Medical Systems, Tochigi, Japan), Micro Vascular Imaging (GE Healthcare, Waukesha, WI, USA), Micro-Flow Imaging (Philips Healthcare, Bothell, WA, USA) and Micro Vascular Flow (Samsung Medison, Seoul, Korea). These techniques appear to improve the detection of slower flow within smaller vessels, increasing the ability to detect subtle vascularity within indeterminate renal masses that were previously below the detection threshold for traditional color and power Doppler techniques.

Leong et al. recently imaged 41 patients harboring 50 renal masses with Superb Micro-Vascular Imaging (SMI). They found that SMI had a higher diagnostic accuracy than standard color Doppler imaging and power Doppler imaging for the detection of vascularity within solid renal masses [47]. They concluded that SMI might have potential in the detection of microvascularity within indeterminate solid renal masses. Subsequently, Mao et al. showed that SMI could distinguish significantly different patterns of vascularization between pathologically proven malignant and benign renal masses in a study on 53 patients [48]. Conversely, conventional Doppler flow imaging could not discern these differences in vascularization.

Although the benefit of these micro-Doppler techniques includes intravenous contrast not being required, there has not been a direct comparison to CEUS for the detection of malignancy, which limits the current applications of these techniques. Future endeavors should specifically study whether the addition of these techniques to CEUS could improve the diagnostic accuracy and could potentially be a useful addition to current techniques in terms of characterizing indeterminate renal masses.

## 4. Conclusions

Ultrasound is a widely available, approachable, and relatively inexpensive imaging modality that allows for real-time evaluation of a suspected renal mass without the drawbacks of ionizing radiation and the risk of an MRI. CEUS has several advantages over traditional grayscale ultrasound in the characterization of indeterminate renal masses. It has a distinct value in the characterization of cystic renal masses and has the potential to differentiate benign from malignant renal masses to some extent. Ultrasound molecular imaging could potentially be an extension of the use of CEUS for serial disease monitoring and longitudinal assessment of treatment response, though it remains in preclinical stages of development at this time. While emerging micro-Doppler techniques and elastography have shown some encouraging applications, current evidence is limited, and neither is ready for widespread clinical use.

## Figures and Tables

**Table 1:** Summary of key findings of contrast-enhanced ultrasound studies.

Study (Year)	Study Type	Type of Renal Mass	Number of Patients (Tumors)	Imaging Type	Key Findings
Furrer et al. (2020)	Systematic review	Solid and cystic renal masses	1290 (1342)	CEUS vs. CECT/CEMRI	CEUS performs at least as well or better than CECT and CEMR in the qualitative diagnosis of benign vs. malignant renal masses.
Yong et al. (2016)	Retrospective cohort study	Indeterminate renal masses in patients with renal impairment	63 (74)	CEUS	CEUS has a high diagnostic performance for the prediction of benignity of renal masses in patients with renal impairment with sensitivity and NPV approaching 100%.
Zhou et al. (2011)	Retrospective cohort study	Solid renal masses with histopathology available or follow-up with MRI	51 (51)	CEUS	CEUS results in good diagnostic confidence for the diagnosis of RCC with a sensitivity of 86% and a specificity of 93%.
Rübenthaler et al. (2018)	Retrospective cohort study	Indeterminate renal masses with histopathology available	255 (255)	CEUS	CEUS resulted in a sensitivity of 99.1% and a sensitivity of 80.5% for the differentiation of being benign vs. malignant.
Lerchbaumer et al. (2020)	Retrospective cohort study	Cystic renal masses	173 (173)	CEUS vs. CECT/CEMRI	CEUS outperforms CECT and CEMRI in the characterization of fine septal and nodular enhancements in cystic renal masses, often leading to an upgrade in Bosniak classification.
Sanz et al. (2016)	Prospective cohort study	Bosniak II–IV cystic renal masses	67 (67)	CEUS vs. CECT	CEUS has a good agreement with CECT regarding the Bosniak classification. Sensitivity and NPV were 100% for the differentiation of benign vs. malignant cystic renal masses.
Ragel et al. (2016)	Prospective cohort study	Cystic renal masses	46 (51)	CEUS vs. CECT	CEUS upstaged cystic renal masses in 31% of cases compared with assessment using CECT.
Defortescu et al. (2017)	Prospective cohort study	Bosniak IIF and III cystic renal masses	47 (47)	CEUS vs. CECT	CEUS outperformed CECT for the differentiation of Bosniak IIF and III cystic renal masses into benign or malignant with a sensitivity of 100%, a specificity of 97% and an NPV of 100%.
Rübenthaler et al. (2016)	Retrospective cohort study	Indeterminate renal masses	36 (36)	CEUS vs. CEMRI	CEUS is useful for the differentiation of benign vs. malignant renal masses, with a sensitivity, specificity and NPV comparable to CEMRI.
Wei et al. (2017)	Retrospective cohort study	Small (<4 cm) renal masses	118 (118)	CEUS vs. CECT	Both CEUS and CECT are effective for the differentiation of benign vs. malignant small renal masses, with a sensitivity for CEUS of 93.5%, a specificity of 68% and an NPV of 73.9%.
Zhang et al. (2019)	Systematic review	Solid and cystic renal masses	NR (2260)	CEUS vs. CECT	CEUS has a higher sensitivity and a comparable specificity for the detection of renal cancer compared with CECT (94% vs. 85% and 77% vs. 75%, respectively).
Zhou et al. (2018)	Systematic review	Cystic renal masses	NR (1142)	CEUS vs. CEMRI	Both CEUS and CEMRI have good diagnostic performance for the differentiation of cystic renal masses in benign vs. malignant renal masses. CEUS has a higher sensitivity, but lower specificity for this diagnosis compared with CEMRI (95% vs. 92% and 84% vs. 91%, respectively).

Studies are listed in the order of mention in the article’s main text. CEUS—contrast-enhanced ultrasound, CECT—contrast-enhanced computed tomography, CEMRI—contrast-enhanced magnetic resonance imaging, NPV—negative predictive value, RCC—renal cell carcinoma, NR—not reported.

**Table 2:** Summary of key findings of elastography studies.

Study (Year)	Study Type	Type of Renal Mass	Number of Patients (Tumors)	Imaging Type	Key Findings
Onur et al. (2015)	Prospective cohort study	Solid renal masses	71 (71)	Strain elastography	Mean strain index values were significantly higher in malignant compared with benign solid renal masses. (Semi-)quantitative analyses of strain elastography may aid in the differentiation of benign and malignant solid renal masses.
Guo et al. (2014)	Retrospective cohort study	Solid renal masses	42 (42)	ARFI	ARFI elastography has a potential value for the differentiation of clear cell RCC vs. pseudotumor or angiomyolipoma vs. pseudotumor but fails to distinguish clear cell RCC and angiomyolipoma.
Keskin et al. (2015)	Prospective cohort study	Renal masses with histopathology available	65 (65)	Strain elastography	(Semi-)quantitative analysis of strain elastography may help in the differentiation of RCC from angiomyolipoma.
Inci et al. (2016)	Prospective cohort study	Solid renal masses, suspicious for malignancy	99 (99)	Strain elastography	(Semi-)quantitative analysis of strain elastography could be useful for the preoperative differentiation of RCC from TCC.
Thaiss et al. (2019)	Prospective cohort study	Small (<4 cm) CECT-indeterminate renal masses	123 (123)	ARFI	ARFI elastography could differentiate clear cell RCC from oncocytoma and chromophobe or papillary RCC.

Studies are listed in the order of mention in the article’s main text. ARFI—acoustic radiation force impulse elastography, RCC—renal cell carcinoma, TCC—transitional cell carcinoma, CECT—contrast-enhanced computed tomography.
